# Molecular Mechanisms Underlying the Higher Prevalence of Anemia in Crohn’s Disease Compared with Ulcerative Colitis: A Systematic Review

**DOI:** 10.3390/ijms27125570

**Published:** 2026-06-20

**Authors:** Dragos-Florin Tesoi, Laura Mihaela Trandafir, Laura Bozomitu, Otilia Elena Frasinariu, Nina Filip, Cornelia Mircea, Monica Hancianu, Oana-Viola Badulescu

**Affiliations:** 1 Grigore T. Popa University of Medicine and Pharmacy, 700115 Iasi, Romania; dragos-florin.tesoi@email.umfiasi.ro (D.-F.T.); laura.bozomitu@umfiasi.ro (L.B.); frasinariu.otilia@umfiasi.ro (O.E.F.); nina.zamosteanu@umfiasi.ro (N.F.); cornelia.mircea@umfiasi.ro (C.M.); monica.hancianu@umfiasi.ro (M.H.); oana.badulescu@umfiasi.ro (O.-V.B.); 2 Emergency Clinical Hospital for Children “Sfanta Maria”, 700309 Iasi, Romania; 3 Hematology Clinic, “St. Spiridon” County Emergency Clinical Hospital, 700111 Iasi, Romania

**Keywords:** Crohn’s disease, ulcerative colitis, inflammatory bowel disease, gut microbiota, siderophores, iron-deficiency anemia, microbiome dysbiosis, lipocalin-2

## Abstract

Anemia represents one of the most frequent systemic complications of inflammatory bowel disease (IBD), with a consistently higher prevalence reported in patients with Crohn’s disease (CD) compared with ulcerative colitis (UC). While chronic inflammation, impaired iron absorption, and intestinal blood loss are recognized contributors, microbiome-mediated mechanisms influencing host iron availability remain insufficiently explored. Emerging evidence indicates that CD-associated dysbiosis is characterized by an increased abundance of siderophore-producing bacteria, particularly members of the Enterobacteriaceae family. Because siderophores are high-affinity iron-chelating molecules capable of competing with host iron acquisition systems and partially escaping lipocalin-2-mediated sequestration, their expansion may contribute to reduced luminal iron bioavailability. In this systematic review, we analyzed comparative microbiome studies published between 2016 and 2026 that directly evaluated microbial differences between CD and UC. CD microbiota consistently demonstrated enrichment in siderophore-associated taxa relative to UC. Based on these findings, we propose that microbiome-driven iron competition may represent an additional mechanistic contributor to the increased prevalence and persistence of anemia observed in CD. Although direct in vivo quantification of siderophore activity in IBD remains limited, the convergence of ecological, functional, and strain-level microbiome evidence supports a biologically plausible interaction between microbial iron-scavenging strategies and host iron metabolism.

## 1. Introduction

### 1.1. Burden of Anemia in Inflammatory Bowel Disease

Anemia is one of the most common extraintestinal complications of IBD, encompassing both CD and UC, and represents a major contributor to morbidity across all age groups. Contemporary evidence identifies anemia as a frequent and clinically relevant comorbidity that reflects both chronic inflammation and nutrient malabsorption inherent to IBD pathophysiology. Recent large-scale analyses indicate that anemia affects a substantial proportion of patients with IBD worldwide. A systematic review and meta-analysis including over 138,000 patients from 22 countries (studies published between 2015 and 2024) reported a pooled prevalence of 36.9% for anemia in the overall IBD population, with iron-deficiency anemia accounting for 32.2% and anemia of chronic disease for 8.1% [1]. These findings confirm that anemia remains highly prevalent despite advances in biologic therapies and disease monitoring. However, prevalence varies widely depending on disease activity, care setting, and geographic region. In adult cohorts, contemporary prospective multicenter data suggest lower but still clinically meaningful rates in well-treated populations. For example, an Italian multicenter observational study reported an anemia prevalence of 13.6% among adults with IBD, with higher rates observed in women of reproductive age (19%) [2]. Anemia rates increase substantially during periods of active disease and at the time of diagnosis, highlighting the dynamic nature of this complication. In pediatric populations, the burden appears even greater, with contemporary cohort studies reporting anemia in approximately one-third to more than half of children at diagnosis, underscoring its early onset and systemic impact [3,4]. Importantly, several large registry-based analyses have demonstrated that anemia prevalence and persistence differ between CD and UC, with CD frequently showing a higher burden, particularly in association with more extensive small-bowel involvement and systemic inflammation [3]. Despite advances in IBD therapy and growing awareness through European Crohn’s and Colitis Organisation (ECCO) recommendations, anemia remains underdiagnosed and undertreated, highlighting a persistent need to better elucidate disease-specific pathogenic mechanisms.

### 1.2. Evidence for a Higher Prevalence of Anemia in CD

Multiple large cohort studies and meta-analyses consistently demonstrate that the burden of anemia differs between CD and UC, with CD generally showing higher rates of both prevalence and incidence. A recent systematic review and meta-analysis reported a pooled prevalence of anemia of 39.3% in CD versus 32.4% in UC, confirming a measurable but clinically meaningful difference between the two entities [1]. These results are supported by population-based data from Sweden showing both a higher incidence (19.3 vs. 12.9 per 100 person-years) and a higher prevalence (28.7% vs. 16.5%) of anemia in CD than in UC [5]. Earlier European individual-patient meta-analysis data also demonstrated a lower likelihood of anemia in UC, reinforcing the concept that disease phenotype influences hematologic complications [6]. Importantly, the difference is already evident at the time of diagnosis. In a large cohort of 1278 newly diagnosed patients, anemia was present in 47% of CD versus 33.8% of UC cases, indicating that divergent mechanisms operate early in disease evolution [7]. A similar pattern is observed in pediatric populations. A large Italian registry including 1634 children with IBD found anemia in 39% of CD versus 33% of UC at diagnosis [3]. This epidemiological asymmetry strongly suggests disease-specific pathogenic drivers, which remain incompletely explained by current models.

### 1.3. Established Mechanisms of Anemia in Crohn’s Disease

Anemia in CD is usually multifactorial, often involving iron deficiency and chronic inflammation. The primary mechanisms are presented in Table 1.

#### 1.3.1. Vitamin B12 and Folate Deficiency in CD

Vitamin B12 (cobalamin) and folate (vitamin B9) are essential micronutrients involved in DNA synthesis and erythropoiesis, and their deficiency can cause megaloblastic anemia, a distinct anemia subtype characterized by macrocytosis and impaired red blood cell production. Vitamin B12 is absorbed in the terminal ileum via an intrinsic factor–dependent process. Because CD frequently affects the terminal ileum and may require surgical resection of this segment, it predisposes patients to impaired B12 absorption. Folate, in contrast, is primarily absorbed in the proximal small intestine (duodenum and jejunum); therefore, inflammation affecting these segments or the use of certain medications (e.g., sulfasalazine, methotrexate) may contribute to folate deficiency [8]. Although most large contemporary cohort studies focus on iron deficiency and inflammatory anemia, there is evidence that vitamin B12 and folate deficiencies are more common in CD than in UC, especially when the disease involves the ileum or follows ileal surgery. One well-characterized retrospective study found that approximately 14.9% of patients with CD had vitamin B12 deficiency compared with 3.2% of patients with UC (*p* = 0.014), and folate deficiency was also more prevalent in Crohn’s patients compared with controls (though differences with UC were less pronounced statistically) [9]. The underlying mechanisms include malabsorption due to ileal inflammation or surgical resection, small-intestinal bacterial overgrowth consuming micronutrients, and increased nutritional requirements during periods of active disease [8,9].

#### 1.3.2. Iron Deficiency Component of Anemia in CD Compared with UC

The pathogenesis of anemia in IBD involves chronic intestinal blood loss, impaired iron absorption, inflammation-mediated iron sequestration, and nutritional deficiencies. Among these mechanisms, iron deficiency remains the dominant contributor to anemia, either alone or combined with anemia of chronic inflammation. Contemporary cohort studies indicate that iron deficiency occurs in approximately a quarter of patients with IBD in routine clinical care, with similar overall prevalence across CD and UC populations, although the underlying mechanisms may differ between the two diseases [10,11]. One of the contributors to anemia in CD is iron deficiency resulting from chronic intestinal blood loss and impaired iron absorption. Mucosal ulcerations and erosions characteristic of active CD lead to ongoing microscopic or overt bleeding, which depletes iron stores over time. In addition, a central mechanism in anemia of chronic disease in CD involves systemic inflammation leading to dysregulated iron metabolism. This process is largely mediated by hepcidin, a peptide hormone produced by the liver that regulates iron availability by controlling the iron exporter ferroportin. Under inflammatory conditions, particularly in active CD, pro-inflammatory cytokines such as interleukin-6 (IL-6) stimulate hepatic production of hepcidin via the JAK/STAT3 signaling pathway, leading to increased hepcidin expression. Elevated hepcidin levels induce internalization and degradation of ferroportin in enterocytes and macrophages, thereby inhibiting iron absorption from the gut and release of recycled iron from macrophages into circulation. Functional iron restriction from this mechanism reduces plasma iron levels available for erythropoiesis even in the presence of adequate or increased iron stores, contributing to anemia of chronic inflammation [12,13,14,15]. In contrast, the pathophysiology of iron deficiency in UC is predominantly related to chronic mucosal blood loss rather than malabsorption. UC is limited to the colonic mucosa and does not directly affect small-intestinal absorptive function. However, the continuous superficial ulceration and friability of the colonic mucosa during active disease lead to recurrent microscopic or overt bleeding, which gradually depletes iron stores [16]. Paradoxically, despite typically more overt bleeding in UC, anemia remains more prevalent in CD cohorts. This apparent discrepancy further suggests that mechanisms beyond simple iron loss are operative in CD.

#### 1.3.3. Enhanced Systemic Inflammation and Hepcidin Upregulation

Chronic inflammation is a central driver of anemia of chronic disease in IBD. One of the most biologically plausible explanations for the excess anemia burden in CD is its typically stronger systemic inflammatory signature. Compared with UC, CD is characterized by transmural inflammation, deeper tissue penetration, and more frequent systemic immune activation. These features are associated with higher circulating pro-inflammatory cytokines, particularly IL-6. Mechanistic work has established IL-6 as the principal inducer of hepatic hepcidin transcription via STAT3 signaling. Because CD is often associated with higher IL-6 activity, patients may experience more pronounced iron sequestration. Nevertheless, studies measuring circulating hepcidin have produced heterogeneous results, and the degree of difference does not consistently match the epidemiological gap in anemia prevalence [15,17,18].

#### 1.3.4. Diagnostic Delay as a Contributing Factor to the Higher Prevalence of Anemia in CD

Several epidemiological studies have reported that anemia is more prevalent in patients with CD than in those with UC, a difference that may be partly explained by the longer diagnostic delay typically associated with CD. The interval between symptom onset and confirmed diagnosis is consistently longer in CD across multiple populations, which may allow prolonged inflammatory activity, intestinal damage, and progressive nutritional deficiencies before treatment initiation.

Systematic reviews analyzing diagnostic delay in IBD have consistently demonstrated that patients with CD experience significantly longer delays compared with those with UC. In a comprehensive systematic review of adult IBD cohorts including more than 30 studies, the median diagnostic delay for UC was reported to be approximately 2–6 months, whereas CD frequently showed longer delays ranging from 2 to 12 months or more. These observations suggest that establishing a diagnosis of CD is inherently more complex due to the heterogeneity of symptoms and the potential involvement of any segment of the gastrointestinal tract [19].

More recent prospective European cohort studies have confirmed these differences. In a multicenter German study including 430 patients with IBD, the total diagnostic delay was significantly longer in CD than in UC (12 months vs. 4 months). The delay was primarily attributed to longer physician diagnostic time, reflecting the more heterogeneous clinical presentation and the need for extensive imaging or endoscopic evaluation to establish a definitive diagnosis [20]. Similar findings have been reported in pediatric populations. A systematic review examining diagnostic delay in pediatric IBD showed that the median time from symptom onset to diagnosis ranged from 4 to 24 months for CD compared with 2 to 18 months for UC, with the majority of studies consistently demonstrating longer delays in CD [21]. Similarly, in a retrospective pediatric study including patients with CD and UC, the overall median diagnostic delay was around 5 months. However, children with CD experienced a significantly longer diagnostic delay than those with UC [22].

The clinical implications of this diagnostic delay are substantial. Prolonged untreated inflammation before diagnosis may contribute to systemic complications, including anemia, which is one of the most common extraintestinal manifestations of IBD. The mechanisms underlying this association are multifactorial. Diagnostic delay in CD allows persistent intestinal inflammation, increased production of pro-inflammatory cytokines such as IL-6 and TNF-α, and prolonged hepcidin-mediated disturbances in iron metabolism. In addition, ongoing mucosal inflammation and small-intestinal involvement may impair iron absorption, while chronic blood loss and systemic inflammatory responses further exacerbate the development of iron deficiency and anemia of chronic disease. Therefore, the longer pre-diagnostic phase typical of CD likely contributes to the higher prevalence and greater severity of anemia observed in these patients compared with those with UC. Taken together, the available evidence suggests that delayed diagnosis is an important clinical factor that may partially explain the higher burden of anemia in CD.

#### 1.3.5. Genetic Regulation of Iron Homeostasis: The Role of *PTPN2* in CD–Associated Anemia

Recent evidence suggests that genetic factors involved in inflammatory signaling may also influence iron metabolism in IBD. One of these factors is the susceptibility gene *PTPN2*, which encodes a protein tyrosine phosphatase that negatively regulates inflammatory signaling pathways, including the JAK–STAT pathway [23]. Proteomic analyses of serum samples from genotyped patients revealed that iron homeostasis was the most significantly downregulated pathway in CD patients carrying the *PTPN2* risk allele, independently of disease activity. Mice lacking functional PTPN2 developed biochemical and hematologic features consistent with anemia, including reduced hemoglobin concentrations, decreased serum iron levels, and diminished tissue iron stores. Radiotracer experiments revealed that these mice also exhibited impaired intestinal iron absorption, accompanied by reduced expression of the apical iron transporter DMT1 in duodenal enterocytes [23].

Taken together, these results identify PTPN2 as a previously underrecognized regulator of systemic iron homeostasis. Because loss-of-function variants in this gene are more strongly associated with CD than with UC, dysregulation of PTPN2-mediated iron handling may represent a genetic mechanism contributing to the higher prevalence of anemia observed in CD [23]. This mechanism is particularly noteworthy because it appears to operate independently of disease activity, suggesting that genetic variation in *PTPN2* could predispose CD patients to iron deficiency and anemia even in the absence of severe intestinal inflammation. Such findings highlight the importance of considering host genetic determinants when investigating the pathophysiology of anemia in IBD.

### 1.4. Classical Mechanisms Do Not Fully Explain Higher Anemia Prevalence in CD

Although several established mechanisms contribute to the development of anemia in CD, they do not fully explain its consistently higher prevalence and persistence compared with UC, suggesting the presence of additional disease-specific determinants. First, ileal involvement, a hallmark feature of CD, is strongly associated with vitamin B12 deficiency due to impaired absorption in the terminal ileum and prior ileal resection. Although B12 deficiency occurs frequently in CD, studies indicate that it accounts for only a minor proportion of anemia cases, with iron-deficiency anemia and inflammation-driven anemia of chronic disease representing the predominant contributors across most patient cohorts [24,25,26]. Moreover, several studies have demonstrated that ileal disease without surgical resection does not consistently increase the risk of clinically significant cobalamin deficiency, suggesting that additional mechanisms contribute to anemia development in CD beyond regional nutrient malabsorption [27]. Furthermore, CD is characterized by more extensive transmural inflammation and greater systemic cytokine signaling than UC, resulting in hepcidin-mediated iron sequestration and reduced intestinal iron absorption, which represent central mechanisms of anemia of inflammation. However, hepcidin-dependent iron restriction is a shared feature across the IBD spectrum and therefore does not sufficiently explain disease-specific differences in anemia prevalence [13]. Recent evidence additionally suggests that genetic susceptibility factors, including variants affecting PTPN2 signaling, may influence epithelial iron transport pathways and contribute to altered iron handling in IBD, although such variants are not universally present and cannot account for population-level differences between CD and UC [23]. In addition, diagnostic delay has been proposed as a contributing factor to the increased severity of nutritional deficiencies in CD. Nevertheless, anemia remains highly prevalent even after diagnosis and during long-term disease monitoring and treatment optimization, indicating that additional mechanisms beyond delayed recognition contribute to persistent iron dysregulation in these patients.

Importantly, epidemiological studies consistently report a higher prevalence of anemia in CD compared with UC despite overlapping inflammatory pathways and treatment strategies, indicating that currently recognized mechanisms—including ileal involvement, vitamin B12 deficiency, systemic inflammatory burden, and host genetic susceptibility—are insufficient to fully explain the differential epidemiology of anemia between these conditions. These observations support the need to investigate additional disease-specific contributors affecting intestinal iron availability, particularly mechanisms operating at the host–microbiota interface.

### 1.5. The Central Idea of a New Mechanism

**Figure 1:**
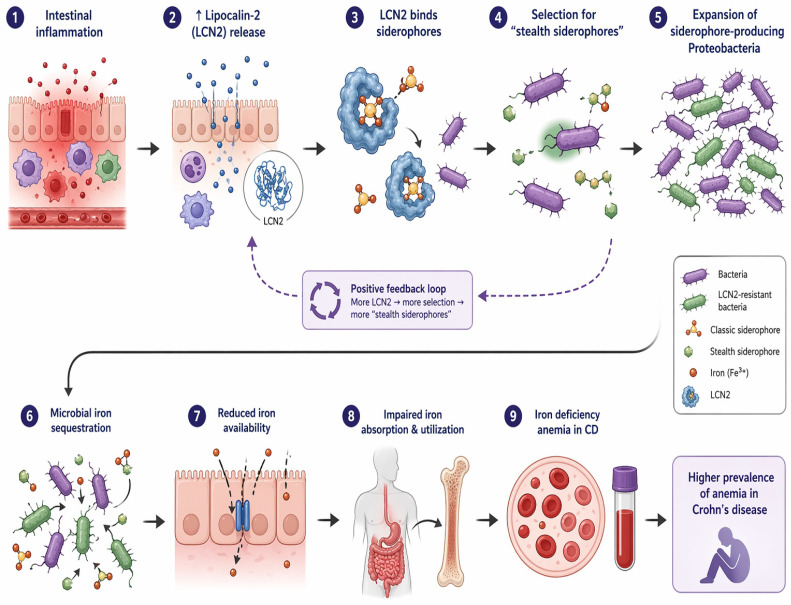
Proposed microbiota-driven mechanism contributing to increased iron deficiency anemia in CD compared with UC. Intestinal inflammation induces epithelial and myeloid release of lipocalin-2 (LCN2), a host siderophore-binding protein involved in nutritional immunity. While LCN2 restricts classical siderophores such as enterobactin, it simultaneously promotes ecological selection of bacteria capable of producing LCN2-resistant (“stealth”) siderophores, including salmochelin, yersiniabactin, aerobactin, and pyoverdine. Expansion of siderophore-producing Proteobacteria increases microbial iron sequestration, reduces luminal iron availability, and may impair both intestinal iron absorption and systemic erythropoiesis. This mechanism may represent an additional microbiome-dependent pathway contributing to the higher prevalence of iron deficiency anemia observed in CD relative to UC.

Based on the limitations of currently recognized mechanisms explaining anemia in CD, we propose that microbial siderophore-mediated iron sequestration may represent an additional mechanism leading to impaired iron availability in these patients. Thus, this pathway may contribute to the higher prevalence of anemia observed in CD compared with UC. Intestinal inflammation in CD is consistently associated with dysbiosis characterized by an expansion of Proteobacteria, including adherent-invasive *Escherichia coli* (AIEC), which possess highly efficient siderophore-based iron acquisition systems such as enterobactin, salmochelin, and yersiniabactin. These bacterial metallophores enable microorganisms to compete for luminal iron under conditions of host-driven nutritional immunity, where iron availability is already restricted by hepcidin-dependent export inhibition and increased expression of iron-binding proteins such as lactoferrin and lipocalin-2. In contrast to UC, CD frequently involves the small intestine—the primary site of dietary iron absorption—thereby increasing the likelihood that microbial iron sequestration directly interferes with epithelial iron uptake at the host–microbiota interface [28,29]. Importantly, intestinal inflammation itself enhances host iron-withholding responses through induction of lipocalin-2 and other antimicrobial proteins that restrict siderophore-dependent iron acquisition; paradoxically, this process selectively favors the expansion of bacteria capable of producing “stealth siderophores” such as salmochelin and yersiniabactin that evade lipocalin-2 binding and remain functionally active within inflamed mucosal environments [30,31]. Such inflammation-driven ecological selection may therefore amplify microbial competition for luminal iron, specifically in CD, where small-intestinal involvement coincides with the principal site of dietary iron absorption. In this context, siderophore-mediated microbial iron capture may further reduce luminal iron availability and represent a mechanistic link between CD–associated dysbiosis and persistent iron deficiency. We therefore hypothesize that enrichment of siderophore-producing microbiota in CD constitutes a disease-specific modifier of intestinal iron bioavailability and may represent a previously unrecognized contributor to the differential epidemiology of anemia between CD and UC (Figure 1).

To further explore this hypothesis, the present study systematically reviews and comparatively analyzes available evidence describing microbiota composition in CD and UC, with particular emphasis on the enrichment of siderophore-producing bacterial taxa across disease phenotypes. By integrating microbiome profiling data with current knowledge of host iron-withholding responses during intestinal inflammation, this work proposes that the preferential expansion of siderophore-producing microorganisms in CD may represent a previously unrecognized mechanism contributing to reduced intestinal iron bioavailability and may help explain the higher prevalence of iron-deficiency anemia observed in CD compared with UC.

## 2. Methods

This study was designed as a systematic literature review to identify sequencing-based comparative studies evaluating differences in gut microbiota composition between patients with CD and UC. The review was conducted in accordance with the Preferred Reporting Items for Systematic Reviews and Meta-Analyses (PRISMA) 2020 guidelines. Study selection was performed independently by two reviewers. Discrepancies between reviewers were resolved through discussion and consensus, and when necessary, a third reviewer was consulted. A structured literature search was performed in three electronic databases—PubMed, Embase (Elsevier), and Scopus—to identify relevant studies published between 1 January 2016 and 1 March 2026. Search strategies combined controlled vocabulary terms and free-text keywords related to CD, UC, gut microbiota, microbiome profiling, dysbiosis, and sequencing-based methodologies (including 16S rRNA sequencing and metagenomics), and were adapted for each database. Searches were restricted to studies involving human subjects and articles published in English. No publication-type restriction was applied in PubMed in order to maximize search sensitivity, whereas searches in Embase and Scopus were limited to original research articles in order to exclude conference abstracts and other non-primary literature. The PubMed search identified 356 records, the Embase search yielded 361 records, and the Scopus search identified 540 records, resulting in a total of 1257 records. Following retrieval, duplicate records were removed using Zotero reference management software (version 9.0.5), resulting in 705 unique articles. These records were subsequently screened by title and abstract, resulting in the exclusion of 620 studies that were either irrelevant to the research question or failed to meet the predefined inclusion criteria, leaving 85 articles for full-text assessment. During full-text screening, an additional 70 articles were excluded due to a lack of direct comparative analysis between CD and UC, absence of sequencing-based microbiota profiling, or other predefined exclusion criteria. Study selection was performed manually without the use of automated screening tools or software-assisted prioritization. Studies were included if they were original research articles conducted in adult and/or pediatric human populations and reported sequencing-based comparative analyses of gut microbiota composition between CD and UC. Only articles published in peer-reviewed journals, in English, between January 2016 and March 2026 were included. Review articles, conference abstracts, editorials, animal studies, in vitro studies, and studies lacking direct microbiota comparison between the two disease entities were excluded. Studies with insufficient methodological detail or unavailable full text were also not considered. No restrictions were applied regarding sample type (fecal samples or mucosal biopsies), patient age group, disease activity status or geographic study location. Following a full-text assessment, 15 studies fulfilled the inclusion criteria and were included in the final qualitative synthesis.

**Figure 2:**
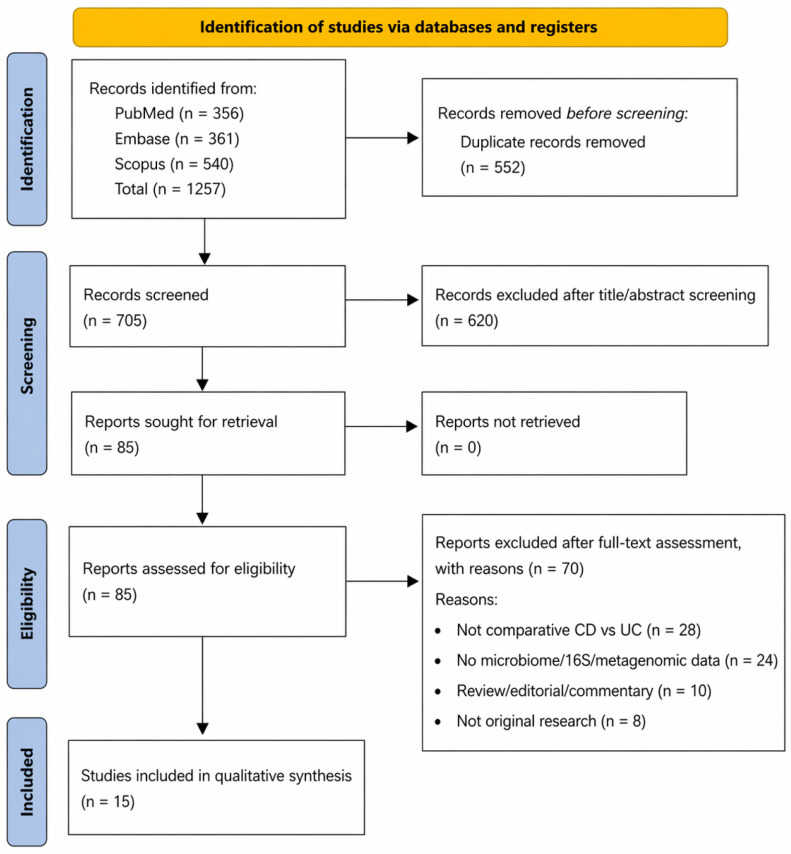
PRISMA 2020 flow diagram of the study selection process. Flow diagram summarizing the identification and selection of sequencing-based comparative studies evaluating differences in gut microbiota composition between CD and UC.

From each included study, the following data were extracted: study design, study population characteristics, age group, biological sample type (fecal samples or mucosal biopsies), microbiota profiling method, sequencing resolution level (genus-, species-, or strain-level, as well as functional profiling), reported differences in gut microbiota composition between CD and UC, and the identification of siderophore-associated taxa enriched in CD. Data extraction was performed manually and independently by two reviewers using a standardized data extraction form. Identified taxa were subsequently evaluated in the context of microbial iron-acquisition mechanisms to support hypothesis generation regarding microbiota-mediated contributions to differences in anemia prevalence between the two inflammatory bowel disease phenotypes. The study selection process, including identification, screening, eligibility assessment, and final inclusion of studies, is summarized in a PRISMA 2020 [32] flow diagram presented in Figure 2. The complete electronic search strategies for all databases, including the exact combinations of controlled vocabulary terms and free-text keywords used, are provided in Table 2 to ensure reproducibility of the literature search.

The methodological quality of included studies was assessed using the Newcastle–Ottawa Scale (NOS), adapted for observational microbiome studies. The assessment evaluated study selection, comparability between CD and UC cohorts, and methodological quality of microbiota profiling approaches. Studies scoring 7–9 points were considered high quality, 5–6 points moderate quality, and ≤4 points low quality. A formal GRADE-based assessment of certainty of evidence was not performed due to the exploratory and mechanistic nature of the review and the heterogeneity of included studies. Thus, we used a structured qualitative framework developed for this review. The evaluation was based on predefined criteria, including sequencing resolution level, taxonomic specificity of reported microbial differences, availability of direct comparisons between CD and UC, and the presence of functional analyses related to iron acquisition pathways. Based on these criteria, studies were categorized into five levels of evidence strength: strong, moderate, limited, neutral, or contradictory. Studies employing shotgun metagenomics or multi-omics approaches with species- or strain-level resolution and consistent findings were considered to provide relatively stronger evidence within the context of this review, whereas studies relying on genus-level 16S rRNA profiling or with limited reporting specificity were classified as moderate or limited evidence. Studies not reporting siderophore-associated taxa were categorized as neutral, while those reporting opposing enrichment patterns were classified as contradictory. This evidence categorization informed the interpretation and synthesis of findings across included studies. A qualitative synthesis approach was applied to integrate findings across studies, focusing on consistent taxonomic and functional differences between CD and UC microbiota, particularly in relation to siderophore-associated bacterial taxa. No statistical synthesis or quantitative pooling of results was performed. The review protocol was not registered in the PROSPERO database because the study was designed as a hypothesis-generating mechanistic synthesis without quantitative effect estimation or planned meta-analysis. Moreover, no standardized effect measures (risk ratios, odds ratios, or mean differences) were applicable, as the included studies primarily reported qualitative and compositional microbiome data without comparable quantitative outcomes. In accordance with PRISMA 2020 guidelines, the completed PRISMA checklist has been included in the Supplementary Materials (Supplementary Table S1) to ensure transparency and reproducibility of the review process. A full list of excluded studies after full-text screening, with reasons for exclusion, is provided in Supplementary Table S2.

## 3. Results and Discussion

Siderophore production represents one of the most efficient microbial strategies for iron acquisition in environments where free iron availability is tightly restricted by host nutritional immunity. A wide range of gut-associated opportunistic pathogens and pathobionts—including members of the Enterobacteriaceae family such as *Escherichia coli*, *Klebsiella* spp., and *Shigella* spp., as well as non-enteric genera such as *Pseudomonas* and *Acinetobacter*—produce high-affinity siderophores that enable them to scavenge ferric iron from host proteins such as transferrin, lactoferrin, and ferritin [33,34]. Because iron is an essential micronutrient required for bacterial respiration, DNA synthesis, and oxidative stress protection, siderophore-mediated uptake systems represent key virulence determinants that enhance bacterial persistence and ecological fitness within the inflamed intestinal niche [33]. Importantly, during intestinal inflammation, host-driven sequestration of iron further intensifies microbial competition for this trace element, promoting the expansion of siderophore-producing taxa capable of overcoming nutritional immunity through high-affinity iron-chelating metabolites [35]. This process not only reshapes microbial community structure but may also contribute to systemic and mucosal iron depletion by diverting luminal iron toward bacterial utilization, thereby exacerbating host iron deficiency [36]. Collectively, these mechanisms support the hypothesis that enrichment of siderophore-producing Proteobacteria represents a biologically plausible link between subtype-specific microbial profiles and altered iron homeostasis in CD compared with UC.

Recent evidence demonstrated that yersiniabactin-producing adherent-invasive *Escherichia coli* promote profibrotic macrophage activation through intracellular zinc sequestration and stabilization of HIF-1α signaling pathways, thereby contributing to intestinal fibrosis and stricture formation in CD [28]. Because fibrostenotic complications are strongly associated with impaired nutrient absorption, chronic mucosal inflammation, and increased risk of iron deficiency, siderophore-mediated microbial activity may indirectly contribute to the development or persistence of anemia in CD patients. In addition to its role as a siderophore, yersiniabactin can also induce intestinal wall fibrosis [28]. This raises the possibility that siderophore-producing Enterobacteriaceae participate not only in the dysregulation of iron homeostasis but also in the structural remodeling of the intestinal wall characteristic of CD. Together, these observations support a model in which siderophore-driven host–microbe iron competition may contribute to both fibrostenotic disease progression and anemia-associated clinical burden.

The present synthesis of metagenomic, 16S rRNA gene sequencing, and multi-omics studies reinforces the concept that CD and UC are characterized by distinct microbial ecosystems, with CD generally exhibiting a stronger enrichment of Proteobacteria and other facultative anaerobic taxa with iron-scavenging capacity. This observation has been proposed as a potential mechanistic contributor to the higher prevalence of anemia in CD compared with UC, potentially mediated through differences in microbial siderophore production and iron sequestration capacity. However, direct comparative studies specifically analyzing differences between CD and UC microbiota remain limited in the current literature. Therefore, this synthesis is based on a selection of 15 studies that provide the most relevant and methodologically comparable data available.

A recent multi-biome shotgun metagenomic study by Akiyama et al. [37] investigated gut microbial, virome, and functional profiles in a well-characterized IBD cohort, including CD, UC, and healthy controls, with external validation across independent international datasets. This approach provides a major advantage over 16S-based studies, as it allows direct identification of clinically relevant pathobionts and their functional potential, including iron-uptake systems. In CD specifically, the authors identified a significant enrichment of multiple opportunistic and siderophore-producing bacteria, including *Escherichia coli*, *Klebsiella pneumoniae*, *Pseudomonas aeruginosa*, *Acinetobacter baumannii*, and *Staphylococcus aureus*, which were not similarly increased in UC. These taxa are well recognized for their capacity to acquire iron through diverse siderophore systems, suggesting a potential ecological advantage in the inflamed intestinal environment of CD. Overall, this study provides strong, multi-cohort evidence supporting a CD–specific expansion of iron-acquisition–competent pathobionts compared with UC. A shotgun metagenomic re-analysis [38] demonstrated that several Enterobacteriaceae species, including *Klebsiella oxytoca*, *Morganella morganii*, and *Citrobacter amalonaticus*, were uniquely enriched in CD compared with UC, while *Escherichia coli* showed a markedly increased abundance across IBD cohorts. Although several siderophore-producing Enterobacteriaceae species, including *Proteus hauseri* and multiple *Citrobacter* taxa, were also enriched in UC, functional pathway analysis demonstrated a significant increase in aerobactin biosynthesis modules specifically in CD samples. These observations suggest that the distinction between CD and UC may not rely solely on the presence of siderophore-producing taxa, but rather on the magnitude and functional activity of iron-acquisition pathways within the microbial community. Additional recent multi-omics analyses by Zheng et al. (2024) further demonstrated that *Escherichia coli* and several *Streptococcus* species are overrepresented in CD compared with UC, supporting the existence of microbiome distinctions between IBD subtypes [39]. Moreover, a study conducted by Kang et al. [40] used whole-metagenome shotgun (WMS) sequencing and linear models for differential abundance (LinDA) to compare fecal microbiomes between CD and UC, followed by validation in an independent cohort. A total of 68 species were differentially abundant between CD and UC. CD was characterized by a higher abundance of pro-inflammatory and Enterobacteriaceae-associated taxa, including *Escherichia coli*, *Shigella dysenteriae*, and multiple *Citrobacter* species, whereas UC showed higher abundance of commensal and putatively anti-inflammatory taxa such as *Prevotella* and *Lachnospira*. At the compositional level, *Escherichia* was markedly increased in CD (9.48% vs. 2.67% in UC), alongside a reduction in butyrate-producing taxa such as *Faecalibacterium*. These differences were partially reproducible in an external cohort (AUC = 0.633), supporting subtype-specific microbiome patterns despite inter-cohort variability. Overall, the findings indicate a stronger expansion of Enterobacteriaceae and inflammatory-associated species in CD compared with UC, consistent with distinct ecological configurations between the two IBD subtypes. This pattern is further supported when considering mucosa-associated microbiota at different intestinal sites, where inflammation-associated ecological niches appear to shape microbial composition. Therefore, a study of site-specific gut microbiota (Schult-Hannemann 2026) demonstrated differential microbial distributions between CD and UC, with mucosal samples in CD showing a relative enrichment of Proteobacteria, including taxa such as *Pseudomonas*, compared with other intestinal sites [41]. This observation is biologically relevant because *Pseudomonas* spp. produce high-affinity siderophores such as pyoverdine, enabling efficient iron sequestration under inflammatory conditions. However, this enrichment was not consistently observed across all sample types, suggesting that the association between *Pseudomonas* and CD may be context-dependent and influenced by mucosal inflammation rather than representing a universal disease-specific signature.

At the level of mucosal surface profiling, similar Proteobacteria-associated patterns have been consistently observed across independent cohorts. An observational cross-sectional mucosa-associated microbiota profiling study (Nishino 2018) analyzed endoscopic brush samples from patients with CD, UC, and non-IBD controls to characterize disease-specific dysbiosis at the intestinal mucosal surface [42]. The authors evaluated microbial community structure and diversity using 16S rRNA gene sequencing from brush-collected mucosal samples across multiple intestinal sites (ileum, cecum, sigmoid colon). The results demonstrated a significant increase in Proteobacteria in CD, accompanied by a marked enrichment of the genus *Escherichia* compared with both UC and healthy individuals. In contrast, UC was characterized by a higher abundance of commensal genera such as *Faecalibacterium*, *Roseburia*, and *Bifidobacterium*. These results point toward a disease-specific microbial divergence between CD and UC, with CD exhibiting a stronger shift toward Enterobacteriaceae-associated taxa [42].

Further supporting these taxonomic and functional shifts, shotgun metagenomic analysis has consistently highlighted *Escherichia coli* as a central driver of CD-associated microbial remodeling. Shotgun metagenomic analysis of a well-characterized Spanish IBD cohort (Serrano Gomez 2021) demonstrated significant species-level differences between CD and UC microbiota, with *Escherichia coli* markedly abundant in CD compared with both UC and healthy controls [43]. Notably, *E. coli* was reported to be almost undetectable in UC samples, highlighting a strong disease-specific microbial signature. Functional pathway analysis further demonstrated that propionate production in CD was primarily driven by Enterobacterales species, whereas *Firmicutes* contributed to this pathway in UC and healthy individuals. Importantly, *E. coli* represented the main contributor to several of the metabolic pathways more frequently identified in CD, supporting its central functional role in disease-associated microbiome remodeling. Collectively, these observations indicate that CD is characterized by both taxonomic and metabolic shifts toward Proteobacteria-dominated microbial communities compared with UC [43]. These observations are further strengthened by integrated multi-omics approaches that combine taxonomic and functional profiling. Integrated metagenomic and metatranscriptomic analyses (Serrano Gomez 2025) identified CD–specific microbiome signatures composed of a panel of 20 microbial species with high diagnostic performance (AUC = 0.94) in external validation cohorts [44]. This multi-omics microbiome study further demonstrated that gut microbial dysbiosis is significantly more pronounced in CD than in UC, both at the taxonomic and functional levels. In particular, *Escherichia coli*, including adherent-invasive strains, was identified as a central species more prevalent in CD and closely linked to disease-associated microbial pathway alterations [44].

Chang et al. also reported compositional differences in the gut microbiota between CD, UC, and healthy controls, including a tendency toward increased abundance of the *Escherichia*–*Shigella* complex in CD within a broader Proteobacteria-associated dysbiosis. However, due to the taxonomic resolution limitations of 16S rRNA profiling, species-level discrimination of *Escherichia coli* and its quantitative comparison between CD and UC remains limited in this cohort [45].

Further support for CD-associated microbial divergence comes from a shotgun metagenomic cohort study (Franzosa et al.), which identified 12 species uniquely enriched in CD, including *Escherichia coli* and *Ruminococcus gnavus*, while UC exhibits a greater expansion of *Bifidobacterium breve* and *Clostridium symbiosum* [46]. These findings support the presence of disease-associated microbial profiles within IBD and are consistent with the enrichment of facultative anaerobic taxa with iron-acquisition capacity in CD microbiomes [46]. Similarly, longitudinal cohort data provide additional evidence for the persistence of CD-associated dysbiosis over time. A longitudinal fecal microbiome cohort study was conducted by Pascal et al. in patients with CD, UC, their healthy first-degree relatives, and unrelated healthy controls, with repeated sampling over 12 months and during disease recurrence [47]. This study showed that CD was more strongly enriched in opportunistic bacteria including *Fusobacterium*, *Escherichia*, *Veillonella*, *Streptococcus*, and *Ruminococcus gnavus* group, while UC shows fewer and less pronounced expansions of such taxa. In addition, Pisani et al. (2022) performed a cross-sectional 16S rRNA gene sequencing study investigating fecal microbiota composition in patients with CD and UC during clinical remission and demonstrated persistent dysbiosis despite inactive disease [48]. At the taxonomic level, CD was more strongly associated with increases in Proteobacteria (especially Enterobacteriaceae), while UC showed higher abundances of *Firmicutes* and *Actinobacteria* [48]. Overall, the article indicates that CD and UC share a core dysbiotic pattern in remission, but still retain distinct microbial signatures, with CD showing more Proteobacteria-driven shifts.

In contrast to these results, several studies provide a more nuanced or partially contradictory perspective. Hansen et al. (2025) conducted a population-based inception cohort study using fecal microbiome profiling and multivariate modeling to compare CD and UC [49]. While both conditions exhibited significant gut microbial dysbiosis relative to controls, including an overall enrichment of Proteobacteria at the IBD level, direct comparisons between CD and UC revealed only taxon-specific differences rather than a consistent phylum-level gradient. Importantly, the study also found that microbiome differences were not only disease-specific but also influenced by inflammation location and other clinical factors, yet CD still tended to exhibit more pronounced dysbiosis compared with UC across most metrics [49]. Similarly, Vermeer et al. performed shotgun metagenomic profiling in therapy-naïve pediatric IBD patients and demonstrated marked microbiome differences between IBD and control groups [50]. However, subgroup analyses comparing CD and UC were limited by sample size and did not identify robust characteristic microbial patterns separating the two conditions. In addition, Dubinsky et al. (2022) performed a strain-resolved comparative genomic study of mucosa-associated *Escherichia coli* isolates from CD and UC cohorts, identifying disease-specific phylogenetic clustering and differences in accessory genome content [51]. Notably, *E. coli* clade III strains, which were more prevalent in UC, carried a higher number of siderophore-associated genes, particularly those related to the yersiniabactin system, and could therefore appear to represent a potential counterargument to the hypothesis of increased siderophore-harboring bacteria in CD [51]. However, this finding reflects differences in siderophore gene carriage at the strain level rather than overall microbiome composition. In contrast, metagenomic studies have consistently demonstrated broader enrichment of multiple siderophore-producing taxa in CD microbiomes, including *Klebsiella*, *E. coli*, and mucosa-associated *Pseudomonas* spp., supporting increased ecosystem-level microbial iron acquisition capacity in CD compared with UC [38,41]. Therefore, while UC–associated clade III *E. coli* strains may exhibit increased siderophore gene load at the strain level, CD microbiomes appear to demonstrate a broader ecosystem-level expansion of multiple iron-scavenging microorganisms, supporting the proposed model of enhanced microbial iron-uptake capacity in CD relative to UC.

When integrating taxonomic, functional, and multi-omics evidence, a consistent pattern emerges. CD is characterized by a more profound expansion of Proteobacteria and facultative anaerobes, many of which possess iron-scavenging systems, including siderophore biosynthesis pathways. UC, in contrast, tends to retain a more stable microbial structure with a relatively higher abundance of commensal *Firmicutes* and *Actinobacteria*, and fewer expansions of bacteria harboring siderophore systems. However, the literature also indicates important heterogeneity across studies, with some strain-level findings and cohort-specific analyses suggesting that siderophore gene content is not preferentially detected in CD. Therefore, while a CD-associated increase in microbial iron acquisition capacity is strongly supported at the ecosystem level, it should be interpreted as a probabilistic and functional trend rather than a universal microbial signature.

To improve transparency and comparability across heterogeneous microbiome study designs, the principal characteristics and key findings of the 15 studies included in this systematic synthesis are summarized in Table 3 and Table 4, with particular emphasis on methodological approaches, study populations, and the relative enrichment of siderophore-associated bacterial taxa in CD compared with UC. Due to the absence of standardized quantitative outcomes across studies, effect estimates and measures of precision were not applicable, and results are presented as qualitative comparisons.

The strength of evidence assigned to each study was evaluated based on sequencing resolution level, taxonomic specificity of reported microbial differences, availability of direct CD versus UC comparisons, and the presence of functional analyses related to iron-harvesting pathways. Studies using shotgun metagenomics or multi-omics approaches with species- or strain-level resolution were considered to provide strong evidence, whereas studies relying on genus-level 16S rRNA profiling were classified as providing moderate or limited evidence depending on cohort design and reporting specificity. Studies not reporting siderophore-associated taxa were classified as neutral, while studies reporting opposite enrichment patterns were categorized as contradictory evidence.

Risk-of-bias assessment using an adapted Newcastle–Ottawa Scale demonstrated that the majority of included studies showed low overall risk of bias, largely due to well-characterized patient cohorts, appropriate microbiome sequencing methodologies, and robust statistical frameworks (Table 5). Multi-cohort metagenomic and longitudinal studies achieved the highest quality scores, reflecting strong comparability between study groups and validated analytical pipelines. Cross-sectional 16S-based studies generally showed moderate risk of bias, primarily due to limited taxonomic resolution and reduced adjustment for potential confounders. The strain-level comparative genomic study by Dubinsky et al. [51] represented a specialized design that limited direct ecological interpretation at the community level but remained methodologically rigorous within its analytical scope. Overall, the included evidence base demonstrates acceptable methodological quality for the synthesis of disease-specific microbiome differences between CD and UC. Due to the absence of quantitative synthesis and the limited number of included studies, the risk of bias due to missing results (reporting bias) was not formally assessed.

### Limitations

A potential limitation of the proposed microbiota-mediated mechanism is the physiological site of iron absorption. In humans, the majority of dietary iron absorption occurs in the proximal small intestine, particularly in the duodenum, where specialized transport systems, such as DMT1 and ferroportin, regulate iron uptake. Under normal physiological conditions, only a limited fraction of dietary iron reaches the distal small intestine and colon, which could theoretically reduce the opportunity for colonic bacteria to compete with the host for luminal iron. From this perspective, the hypothesis that siderophore-producing bacteria contribute to iron restriction and anemia in CD may initially appear difficult to reconcile with the well-established physiology of intestinal iron absorption [52]. However, several pathophysiological features of IBD challenge this simplified view of intestinal iron distribution. First, inflammation-associated upregulation of hepcidin markedly reduces ferroportin-mediated iron export from enterocytes, thereby limiting net iron absorption despite normal dietary intake. As a consequence, iron can accumulate within enterocytes and may subsequently be lost into the intestinal lumen during epithelial turnover, effectively increasing luminal iron availability in more distal intestinal segments [53]. Second, intestinal inflammation itself profoundly alters epithelial barrier function and nutrient absorption. In CD, where inflammation frequently involves the small intestine, mucosal damage, villous architectural distortion, and inflammatory cytokine signaling can reduce the efficiency of iron uptake in the proximal intestine. Consequently, a greater proportion of luminal iron may escape absorption and progress toward the distal gut [53]. Furthermore, several studies suggest that luminal iron availability in the colon is not negligible even under physiological conditions. Dietary iron that remains unabsorbed, iron derived from epithelial cell turnover, and iron released from intestinal bleeding can all contribute to the luminal iron pool accessible to the gut microbiota. Importantly, the colonic microbial community possesses highly efficient iron-sequestration systems, including siderophores such as enterobactin and yersiniabactin, which enable bacteria to sequester iron even when it is present at extremely low concentrations [54,55]. Although bacterial density is lower in the proximal small intestine compared with the colon, several studies have demonstrated that microbial dysbiosis in CD extends to the upper gastrointestinal tract, including the stomach and duodenum. Alterations in duodenal microbial composition have been reported even in patients in clinical remission, suggesting persistent proximal intestinal dysbiosis [56].

On the other hand, direct clinical evidence linking siderophore production within the intestinal microbiota to systemic iron deficiency in patients with inflammatory bowel disease remains limited. While numerous bacterial species possess highly efficient iron-scavenging systems, including siderophores such as enterobactin and yersiniabactin, most currently available data derive from experimental microbiology studies or mechanistic host–microbe interaction models rather than clinical investigations specifically addressing iron metabolism in IBD populations. Consequently, the extent to which bacterial iron sequestration directly influences host iron availability and erythropoiesis in vivo remains incompletely understood.

In addition, host defense mechanisms such as lipocalin-2 are known to restrict bacterial siderophore-dependent iron acquisition and might therefore be expected to limit the biological relevance of this pathway. Importantly, several Enterobacteriaceae associated with CD produce modified “stealth siderophores,” including salmochelin and yersiniabactin, which evade lipocalin-2 binding and remain functional within inflamed mucosal environments, suggesting that bacterial iron-uptake systems can remain active despite host antimicrobial countermeasures [57,58,59].

Another important limitation of the current evidence base is the absence of studies directly quantifying siderophore production or iron-acquisition activity of the gut microbiota in comparative CD and UC cohorts. As a result, the proposed contribution of siderophore-mediated microbial competition for luminal iron to differences in anemia prevalence between the two disease phenotypes should be regarded as hypothesis-generating rather than experimentally validated. Moreover, clinical heterogeneity among included populations, including differences in disease activity status, treatment exposure, disease duration, and age group (adult versus pediatric cohorts), may further influence microbiota composition and limit the generalizability of observed microbial signatures. An additional limitation is that none of the studies included in the present review simultaneously evaluated microbiota composition and detailed iron metabolism parameters within the same patient cohorts. Consequently, direct associations between the abundance of siderophore-producing taxa and clinically relevant markers such as serum iron, transferrin saturation, ferritin, hepcidin, or hemoglobin levels remain largely unexplored. The proposed link between microbiota-derived iron acquisition mechanisms and anemia therefore relies on the integration of separate lines of evidence rather than on direct patient-level correlations. Future prospective studies combining microbiome sequencing with comprehensive iron metabolism phenotyping will be essential to determine whether enrichment of siderophore-producing microorganisms is independently associated with iron deficiency severity and anemia outcomes in CD.

Finally, due to methodological variability and outcome heterogeneity across studies, a quantitative meta-analysis was not feasible, and the present review therefore provides a qualitative synthesis of currently available sequencing-based comparative evidence.

Despite these limitations, the siderophore hypothesis remains intriguing because it introduces a previously underexplored dimension of host–microbiota interaction in iron metabolism. The possibility that microbial iron-scavenging capacity contributes to host iron restriction highlights the need for integrative studies combining metagenomics, microbial functional profiling, and detailed characterization of iron metabolism in patients with IBD. Such approaches may help determine whether specific siderophore-producing taxa are enriched in CD and whether their abundance correlates with iron deficiency and anemia severity.

An alternative, non-mutually exclusive explanation should also be considered. While the present hypothesis proposes that expansion of siderophore-producing bacteria may contribute to host iron restriction, the opposite direction of causality may also occur. Iron deficiency and inflammation-driven hypoferremia are known to create an iron-limited intestinal environment that could selectively favor bacterial taxa possessing highly efficient iron-acquisition systems. Under this model, microbial enrichment for siderophore-producing organisms would represent not only a cause but also a consequence of disturbed iron homeostasis. It is therefore conceivable that a bidirectional feedback loop exists, whereby inflammation-induced iron restriction promotes expansion of siderophore-producing bacteria, which in turn further intensifies competition for luminal iron. Longitudinal studies integrating microbiome profiling with dynamic assessment of iron metabolism parameters will be required to clarify the temporal and causal relationships underlying this interaction.

## 4. Conclusions

If confirmed, the proposed siderophore-mediated microbial iron sequestration model may have several important clinical and therapeutic implications for understanding and managing anemia in CD. Current treatment strategies for iron-deficiency anemia in IBD primarily focus on iron replacement therapy—either oral or intravenous—combined with control of intestinal inflammation, as recommended by international guidelines, such as those issued by ECCO. However, despite optimized therapy, iron deficiency frequently persists or recurs in patients with CD, suggesting that additional mechanisms limiting intestinal iron bioavailability may remain insufficiently addressed by existing treatment paradigms.

Within this context, the identification of microbiota-driven competition for luminal iron as a potential disease-specific contributor to anemia raises the possibility that modulation of siderophore-producing bacterial communities could represent a novel adjunctive therapeutic strategy. Experimental studies have demonstrated that inflammation-associated expansion of siderophore-producing Enterobacteriaceae—including adherent-invasive *Escherichia coli*—is promoted by host iron-withholding responses, such as lipocalin-2 production, which selectively favors bacterial strains capable of synthesizing “stealth siderophores” that evade host antimicrobial sequestration mechanisms [60]. This evidence suggests that microbial iron-harvesting pathways represent a biologically relevant therapeutic target at the host–microbiota interface.

From a translational perspective, several emerging strategies could theoretically be adapted to influence siderophore-mediated microbial iron competition. These include microbiota-directed therapies such as targeted antibiotic approaches against AIEC strains, probiotic or ecological restoration strategies aimed at reducing Proteobacteria expansion, and dietary interventions designed to modify luminal iron availability and microbial iron utilization patterns.

In addition, advances in the understanding of host nutritional immunity raise the possibility that biomarkers reflecting siderophore activity or lipocalin-2–mediated antimicrobial responses could serve as indicators of microbiota-driven iron competition in CD. Fecal lipocalin-2 has already been proposed as a sensitive biomarker of intestinal inflammation and microbial ecological shifts in IBD and has the potential to represent a candidate surrogate marker for evaluating host–microbiota interactions affecting iron availability [1].

Finally, the proposed host–microbiota competition model for luminal iron suggests that persistent iron deficiency in CD may not always reflect inadequate supplementation or uncontrolled inflammation alone, but could also involve disease-specific microbial determinants of iron bioavailability. Recognition of this mechanism could therefore support the development of microbiota-informed therapeutic strategies and contribute to more personalized management approaches for anemia in CD. In conclusion, the proposed contribution of siderophore-mediated iron sequestration to the higher prevalence of anemia observed in CD represents a biologically plausible but insufficiently explored mechanism. Future studies integrating shotgun metagenomics, metatranscriptomics, metabolomics, and targeted siderophore quantification assays in well-characterized CD and UC cohorts are therefore needed to clarify whether disease-specific microbiome functional profiles translate into measurable differences in microbial iron-uptake activity in vivo. Clarifying this host–microbiota interaction may therefore improve our understanding of why anemia remains disproportionately common in CD despite advances in anti-inflammatory and iron-replacement therapies.

## Figures and Tables

**Table 1:** Mechanisms of Anemia in CD.

Mechanism	Primary Cause	Pathophysiology
Iron-deficiency anemia	Chronic blood loss	Ulceration of the intestinal mucosa leads to continuous blood loss, exhausting iron stores.
	Malabsorption	Active inflammation in the duodenum or proximal jejunum, or surgical resection, reduces dietary iron absorption.
	Dietary Intake	Reduced appetite and dietary restrictions common during active disease
Anemia of chronic disease	Hepcidin elevation and iron sequestration	Inflammation increases hepatic synthesis of hepcidin. Hepcidin binds to ferroportin in enterocytes and macrophages, leading to its degradation. This blocks iron absorption and traps iron inside macrophages, making it unavailable for erythropoiesis.
	Myelosuppression	Inhibition of bone marrow erythropoiesis by inflammatory cytokines
Other causes	Vitamin Deficiency	Deficiency in vitamin B12 or folic acid, commonly due to ileal resection or inflammation of the ileum or duodenum, resulting in macrocytic changes.
	Drug-induced anemia	Medications, such as sulfasalazine, can affect folate levels; others, such as azathioprine, may cause aplasia.

**Table 2:** Literature search strategy.

Database	Search String	Filters Applied	Date of Search	Results Retrieved
PubMed	((“Crohn Disease”[Mesh] AND “Colitis, Ulcerative”[Mesh]) OR ((“Crohn disease” OR “Crohn’s disease”) AND (“ulcerative colitis” OR UC))) AND (microbiota OR microbiome OR dysbiosis OR “gut microbiota”) AND (16S OR metagenomic* OR sequencing OR profiling)	Humans; English; Adults + Pediatric; Publication date: Jan 2016–Mar 2026	March 2026	356
Embase (Elsevier)	(‘crohn disease’/exp AND ‘ulcerative colitis’/exp) AND (‘gut microbiota’/exp OR microbiota OR microbiome OR dysbiosis) AND (‘16s rrna’/exp OR metagenomic* OR sequencing OR profiling OR ‘microbiome analysis’)	Humans; English; Article type only; Publication date: Jan 2016–Mar 2026	March 2026	361
Scopus	TITLE-ABS-KEY(((“Crohn disease” OR “Crohn’s disease”) AND (“ulcerative colitis” OR UC)) AND (microbiota OR microbiome OR dysbiosis OR “gut microbiota” OR “intestinal microbiota”) AND (16S OR metagenomic* OR sequencing OR profiling OR “shotgun sequencing”))	Article type only; Humans; English; Publication date: Jan 2016–Mar 2026	March 2026	540

**Table 3:** Characteristics of included studies.

Study	Year	Study Type	Population	Sample Type	Method	Sequencing Resolution Level
Akiyama et al. [37]	2024	Multi-biome multi-omics cohort	Adult IBD cohort with external validation	Stool	Shotgun metagenomics + virome + functional profiling	Species + functional
Khorsand et al. [38]	2022	Dataset re-analysis (IBDMDB)	Multi-cohort dataset	Stool	Shotgun metagenomics + pathway analysis	Functional pathway-level
Zheng et al. [39]	2024	Multi-cohort diagnostic metagenomics study	International adult cohorts	Stool	Shotgun metagenomics + MaAsLin2 modeling	Species-level
Kang et al. [40]	2023	Comparative microbiome modeling study	Adult CD vs. UC cohorts	Stool	Whole-metagenome sequencing + LinDA	Species-level
Schult-Hannemann et al. [41]	2026	Site-specific microbiota study	Adult IBD cohort	Mucosal biopsies	16S profiling across intestinal locations	Genus-level
Nishino et al. [42]	2018	Cross-sectional mucosa-associated microbiota study	Adult CD, UC, controls	Endoscopic brush samples	16S rRNA sequencing	Genus-level
Serrano-Gómez et al. [43]	2021	Shotgun metagenomic cohort study	Spanish IBD cohort	Stool	Shotgun metagenomics	Species-level
Serrano-Gómez et al. [44]	2025	Integrated multi-omics biomarker study	Multi-cohort CD vs. UC dataset	Stool	Metagenomics + metatranscriptomics	Functional (expression-level)
Chang et al. [45]	2021	Cross-sectional fecal microbiota study	Taiwanese IBD cohort	Stool	16S rRNA sequencing + LEfSe	Genus-level
Franzosa et al. [46]	2019	Multi-omics microbiome structure/function study	Adult IBD cohort	Stool	Metagenomics + metabolomics	Species + functional
Pascal et al. [47]	2017	Longitudinal microbiome cohort study	CD, UC, relatives, controls	Stool	Longitudinal 16S profiling	Genus-level
Pisani et al. [48]	2022	Cross-sectional remission-phase microbiome study	CD and UC in remission	Stool	16S rRNA sequencing	Genus-level
Hansen et al. [49]	2025	Population-based inception cohort	Adult IBD cohort	Stool	Fecal microbiome profiling + multivariate modeling	Species-level
Vermeer et al. [50]	2026	Pediatric therapy-naïve metagenomic study	Pediatric de novo IBD	Stool	Shotgun metagenomics	Species-level
Dubinsky et al. [51]	2022	Strain-resolved comparative genomic study	Adult mucosa-associated isolates	Mucosa-associated *E. coli* strains	Comparative genomics	Strain-level

**Table 4:** Microbiome differences between CD and UC with relevance to siderophore-associated taxa.

Study	CD vs. UC Microbiota Differences	Siderophore-Associated Taxa	Evidence Strength
Akiyama et al. [37]	Opportunistic pathogens enriched in CD vs. UC	*Escherichia coli*, *Klebsiella pneumoniae*, *Pseudomonas aeruginosa*, *Acinetobacter baumannii*, *Staphylococcus aureus*	Strong
Khorsand et al. [38]	Enterobacteriaceae and aerobactin biosynthesis enriched in CD	*K. oxytoca*, *M. morganii*, *C. amalonaticus*, *P. hauseri*, *E. coli*	Strong
Zheng et al. [39]	*E. coli* enriched in CD but not UC	*E. coli*	Moderate
Kang et al. [40]	Enterobacteriaceae enriched in CD vs. UC	*E. coli*, *Shigella dysenteriae*, *Citrobacter* spp.	Moderate
Schult-Hannemann et al. [41]	Increased mucosal Proteobacteria in CD	*Pseudomonas* spp.	Limited
Nishino et al. [42]	Proteobacteria enriched in CD vs. UC	*Escherichia* genus	Moderate
Serrano-Gómez et al. (2021) [43]	*E. coli* strongly enriched in CD vs. UC	*E. coli*	Strong
Serrano-Gómez et al. (2025) [44]	CD dysbiosis more pronounced vs. UC	*E. coli* (central species)	Strong
Chang et al. [45]	Increased *Escherichia*–*Shigella* complex in CD	*Escherichia*/*Shigella* complex	Moderate
Franzosa et al. [46]	Species-level ecological differences between CD and UC	*E. coli*	Strong
Pascal et al. [47]	Pathobionts enriched in CD vs. UC	*Escherichia* genus	Moderate
Pisani et al. [48]	Proteobacteria enriched in CD vs. UC	Enterobacteriaceae (family-level)	Limited
Hansen et al. [49]	Differences influenced by phenotype and disease location	Not specifically reported	Neutral
Vermeer et al. [50]	Limited CD vs. UC subgroup separation	Not specifically reported	Neutral
Dubinsky et al. [51]	Higher siderophore gene load in UC clade III strains	Yersiniabactin-associated genes	Contradictory (strain-level)

**Table 5:** Risk of bias assessment using the Newcastle–Ottawa Scale (adapted for microbiome observational studies).

Study	Selection (4)	Comparability (2)	Outcome (3)	Total Score	Risk
Akiyama et al. [37]	4	2	3	9	Low
Khorsand et al. [38]	3	1	3	7	Low
Zheng et al. [39]	4	2	3	9	Low
Kang et al. [40]	3	2	3	8	Low
Schult-Hannemann et al. [41]	3	1	3	7	Low
Nishino et al. [42]	3	1	3	7	Low
Serrano-Gómez et al. (2021) [43]	3	2	3	8	Low
Serrano-Gómez et al. (2025) [44]	4	2	3	9	Low
Chang et al. [45]	3	1	2	6	Moderate
Franzosa et al. [46]	4	2	3	9	Low
Pascal et al. [47]	4	2	3	9	Low
Pisani et al. [48]	3	1	2	6	Moderate
Hansen et al. [49]	4	2	3	9	Low
Vermeer et al. [50]	3	1	2	6	Moderate
Dubinsky et al. [51]	2	1	3	6	Moderate

## Data Availability

No new data were created or analyzed in this study. Data sharing is not applicable to this article.

## Supplementary Materials

The following supporting information can be downloaded at: https://www.mdpi.com/article/10.3390/ijms27125570/s1.

## Abbreviations

The following abbreviations are used in this manuscript:

AIEC	Adherent-invasive *Escherichia coli*
CD	Crohn’s disease
ECCO	European Crohn’s and Colitis Organisation
FMT	Fecal microbiota transplantation
IBD	Inflammatory bowel disease
IL	Interleukin
LEfSe	Linear discriminant analysis effect size
MaAsLin2	Multivariate Association with Linear Models 2
NGS	Next-generation sequencing
NOS	Newcastle–Ottawa Scale
PRISMA	Preferred reporting items for systematic reviews and meta-analyses
ROS	Reactive oxygen species
TNF	Tumour necrosis factor
UC	Ulcerative colitis
WGS	Whole-genome sequencing
WMS	Whole-metagenome sequencing
