# Supplementary material for: Molecular Mechanisms Underlying the Higher Prevalence of Anemia in Crohn’s Disease Compared with Ulcerative Colitis: A Systematic Review
Source: Int J Mol Sci. 2026 Jun 20;27(12):5570. doi: 10.3390/ijms27125570 (PMC13300524; doi:10.3390/ijms27125570)
Supplement: Supplementary file 1 [file ijms-27-05570-s001.zip › Supplementary Table S2.pdf]

No.	Reference	Reason for exclusion
1	Zheng et al., 2022	Review article (not original research)
2	Zakharzhevskaya et al., 2025	Does not provide a direct comparative analysis between CD and UC
3	Masoodi et al., 2019	No direct comparison between CD and UC
4	Seyed Tabib et al., 2020	Review article (not original research)
5	de Meij et al., 2018	No direct comparison between CD and UC
6	Davidson et al., 2025	Does not use sequencing-based gut microbiota profiling
7	Ryvchin et al., 2021	No direct comparison between CD and UC
8	Han et al., 2024	No direct comparison between CD and UC
9	Alsulaiman et al., 2023	No direct comparison between CD and UC
10	Joo & Nam, 2024	No direct comparison between CD and UC
11	Li et al., 2019	Includes only Crohn’s disease (no UC comparison)
12	Henry et al., 2022	Review article (not original research)
13	Aldrian et al., 2025	Not original research (meta-analysis)
14	Čipčić Paljetak et al., 2022	No direct comparison between CD and UC
15	Kiernan et al., 2019	Does not analyze intestinal microbiota
16	Manandhar et al., 2021	No direct comparison between CD and UC
17	Chen et al., 2025	Review article (not original research)
18	Vestergaard et al., 2024	Review article (not original research)
19	DeSantis et al., 2025	No direct comparison between CD and UC
20	Gonzalez et al., 2022	Includes only Crohn’s disease (no UC comparison)
21	Oliveira et al., 2023	Review article (not original research)
22	Orejudo et al., 2025	No direct comparison between CD and UC
23	Hu et al., 2023	No direct comparison between CD and UC
24	Nishida et al., 2021	Review article (not original research)
25	Aldars-García et al., 2021	Review article (not original research)
26	Tyler et al., 2016	No direct comparison between CD and UC
27	Weng et al., 2019	No direct comparison between CD and UC
28	Li et al., 2024	No direct comparison between CD and UC
29	Lacroix et al., 2021	Review article (not original research)
30	Vester-Andersen et al., 2019	Includes only Crohn’s disease (no UC comparison)
31	Ma et al., 2018	No direct comparison between CD and UC
32	Arribas-Rodríguez et al., 2025	No direct comparison between CD and UC
33	Liu et al., 2022	Does not involve primary sequencing-based microbiota profiling
34	Sezgin et al., 2022	Not sequencing-based microbiota profiling
35	Forbes et al., 2018	No direct comparison between CD and UC
36	Borren et al., 2021	No direct comparison between CD and UC
37	Dheer et al., 2020	No direct comparison between CD and UC
38	Cimická et al., 2022	No direct comparison between CD and UC
39	Masoodi et al., 2019	No direct comparison between CD and UC
40	Soltys et al., 2020	No direct comparison between CD and UC
41	Toto et al., 2024	No direct comparison between CD and UC
42	Ryan et al., 2020	No direct comparison between CD and UC
43	Al-Amrah et al., 2023	No direct comparison between CD and UC
44	Park et al., 2025	Uses culture-based methods, not sequencing-based microbiota profiling

45	Dahal et al., 2023	Focus on bacterial isolates and antimicrobial susceptibility, not gut microbiota profiling
46	Ding et al., 2026	General microbiome restructuring in IBD; no clear CD vs UC comparative sequencing analysis
47	Vega et al., 2022	No direct comparison between CD and UC
48	Zdanowicz et al., 2025	No direct comparison between CD and UC
49	Zhang et al., 2025	Review-type article (conceptual discussion of dysbiosis)
50	Beca et al., 2026	No direct comparison between CD and UC
51	Knoll et al., 2017	No direct comparison between CD and UC
52	Qi et al., 2021	Oral microbiota study (not gut CD vs UC comparison)
53	Yu et al., 2025	Machine learning biomarker signature without CD vs UC comparative analysis
54	Lo Sasso et al., 2021	No direct comparison between CD and UC
55	Wang et al., 2024	No direct comparison between CD and UC
56	Ma et al., 2024	General comparative IBD analysis without CD vs UC separation
57	Luo & Yang, 2025	Pediatric biomarker prediction study, not CD vs UC comparative sequencing study
58	Lo Presti et al., 2023	Focus on Prevotella copri in IBS and IBD, not CD vs UC comparison
59	Shome et al., 2022	Serological antibody profiling, not microbiota sequencing comparison between CD and UC
60	Glassner et al., 2020	Narrative review
61	Guzzo et al., 2022	Review article (fungi/protozoa/bacteriophages), not primary CD vs UC microbiome study
62	Abdelbary et al., 2022	No direct comparison between CD and UC
63	Li et al., 2026	PCR-based diagnostic/monitoring study; not sequencing-based CD vs UC comparative microbiome profiling
64	Shah et al., 2025	Systematic review (secondary literature; no original CD vs UC sequencing data)
65	Alzahrani et al., 2025	No direct comparison between CD and UC
66	Vatn et al., 2025	No direct comparison between CD and UC
67	Rausch et al., 2025	No direct comparison between CD and UC
68	Abdel-Rahman & Morgan, 2022	Systematic meta-analysis (secondary synthesis; no original comparative sequencing data)
69	Mirsepasi-Lauridsen et al., 2018	No direct comparison between CD and UC
70	Aabrekk et al., 2025	Cohort-based prognostic microbiome study in IBD without explicit CD vs UC comparison
